# Time-course differential lncRNA and mRNA expressions in radioresistant hypopharyngeal cancer cells

**DOI:** 10.18632/oncotarget.17343

**Published:** 2017-04-21

**Authors:** Jieyu Zhou, Shengda Cao, Wenming Li, Dongmin Wei, Zhentao Wang, Guojun Li, Xinliang Pan, Dapeng Lei

**Affiliations:** ^1^ Department of Otorhinolaryngology, Qilu Hospital, Shandong University, Key Laboratory of Otolaryngology, NHFPC - Shandong University, Jinan, Shandong, 250012, P.R. China; ^2^ Department of Otorhinolaryngology, Shanghai Ninth People's Hospital, Shanghai Jiaotong University, School of Medicine, Shanghai, 200011, P.R. China; ^3^ Department of Head and Neck Surgery, The University of Texas MD Anderson Cancer Center, Houston, TX, 77030, USA; ^4^ Department of Epidemiology, The University of Texas MD Anderson Cancer Center, Houston, TX, 77030, USA

**Keywords:** hypopharyngeal squamous cell carcinoma, radioresistance, lncRNA, mRNA, microarray

## Abstract

Radioresistance remains a major problem in the treatment of patients with hypopharyngeal squamous cell carcinoma (HSCC). Long noncoding RNAs (lncRNAs) have important roles in the development, invasion, and metastasis of various tumors, including HSCC, but little is known about the role of lncRNAs in cancer radioresistance. The aim of this study was to identify radioresistance-related lncRNAs and mRNAs in radioresistant (RS) hypopharyngeal cancer subclone RS-FaDu cells. In this study, we performed microarray analysis to find the differences in time-course lncRNA and mRNA expression profiles between RS-FaDu and parent FaDu cells after 4 Gy radiation therapy, whose reliability was confirmed by validation experiment. Among these consistently dysregulated lncRNAs, we found that some lncRNAs (e.g., TCONS_00018436) might control resistance of HSCC cells to radiation. Furthermore, our bioinformatics analyses from mRNA/lncRNA microarray data showed that certain lncRNAs or mRNAs potentially are involved in radioresistance of HSCC. Our results from this study laid the foundation for further investigating the roles of these lncRNAs and mRNAs as promising candidates in the occurrence and development of HSCC radioresistance.

## INTRODUCTION

Hypopharyngeal squamous cell carcinoma (HSCC), which originates from the mucosa of the hypopharynx, has one of the poorest prognoses among head and neck cancers [1]. Currently, the standard treatment strategy for HSCC is surgery followed by radiotherapy [1]. Significant advances in radiotherapeutic strategies for HSCC, such as intensity-modulated radiotherapy (IMRT), image-guided radiotherapy (IGRT), and helical tomotherapy (TOMO), have been made in recent years [1, 2]. However, local recurrence and distant metastases after radiotherapy due to tumor radioresistance remain a serious obstacle to successful treatment of HSCC, and the 5-year survival rate remains at approximately 25% to 40% [3]. Although mechanisms of radioresistance have been extensively studied [4, 5], the underlying molecular pathways and targets involved in HSCC radioresistance are not fully understood. Currently, there are few strategies available for overcoming this clinical problem.

In the past decade, advances in genome-wide analysis of gene expression have revealed that the majority of genes in the genome are transcribed into non-coding RNAs (ncRNAs) [6]. Long non-coding RNAs (lncRNAs) are ncRNAs longer than 200 nucleotides [7], and they have important roles in chromatin modification and transcriptional and post-transcriptional processing [8, 9]. Specifically, lncRNAs have been demonstrated to promote the development, invasion, and metastasis of many tumors by a variety of mechanisms [10, 11]. Notably, several studies have shown that lncRNAs are extremely important for controlling cancer radioresistance [12–15], but the roles of lncRNAs in HSCC radioresistance are still unknown.

Accumulating evidence demonstrates that lncRNAs are widely involved in the regulation of proliferation, DNA damage response, apoptosis, and the cell cycle in cancer cells [16–22], all of which are closely associated with the development of radioresistance [23]. Moreover, the roles of several lncRNAs, such as MALAT1 [13, 24], TUG1 [14], NEAT1 [25], and BOKAS [26], in radioresistance have been identified, although their detailed mechanisms remain largely unclear. Radioresistance is the leading cause of recurrence and poor prognosis in HSCC patients. Hence, it is of vital significance to figure out whether or not lncRNAs can become biomarkers for radioresistant HSCC and explore the molecular mechanisms underlying HSCC radioresistance.

It is very unlikely that a single molecule or gene is responsible for radioresistance in HSCC; therefore, to provide useful information for elucidating the molecular mechanisms that lncRNAs and mRNAs are involved in HSCC radioresistance, we used microarray techniques to perform large-scale analyses of lncRNA and mRNA expressions to comprehensively search for mechanisms of HSCC radioresistance. We initially generated a radioresistant HSCC subclone (RS-FaDu) from the parental FaDu cell line via long-term fractionated irradiation. Subsequently, we investigated differences in time-course lncRNA and mRNA expression profiles between RS-FaDu and parent FaDu cells after radiation therapy by microarray and bioinformatics analyses.

## RESULTS

### Establishment and validation of radioresistant HSCC subclone cell line

**Figure 1:**
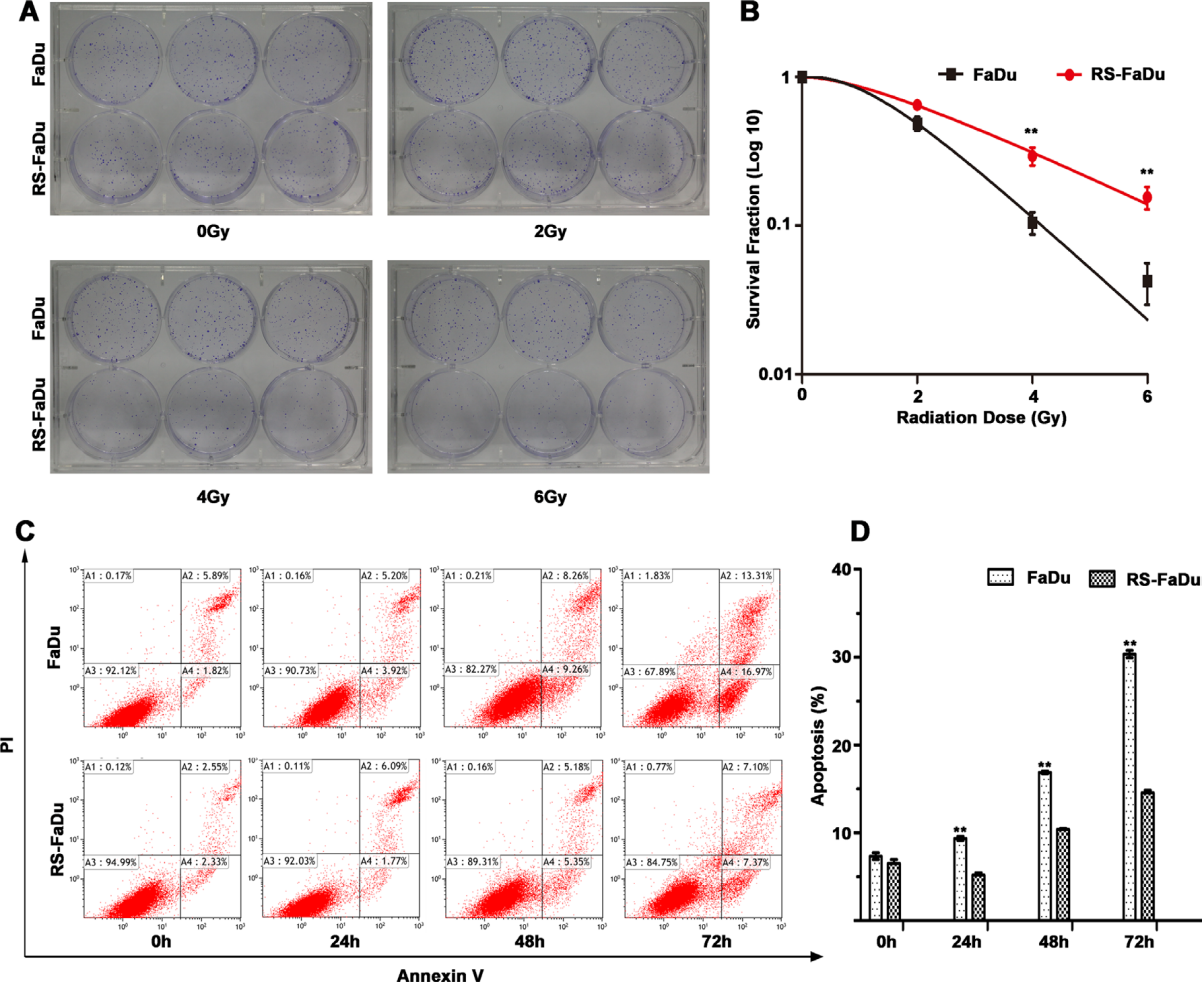
Radioresistance measurement by clonogenic survival assay and apoptosis assay (**A**) RS-FaDu and parental FaDu cells irradiated with different radiation doses (0, 2, 4, and 6 Gy) and the crystal violet-stained colonies were photographed at 12 days after irradiation. (**B**) Colonies containing more than 50 cells for survival colonies and scoring. (**C**) RS-FaDu and FaDu cells irradiated with 4 Gy. The apoptosis detection by FCM Annexin V/PI staining. The proportions of Annexin V+/PI− and Annexin V+/PI+ cells for early- and late-stage apoptosis. (**D**) At 0 h after irradiation with 4 Gy, there was no difference between RS-FaDu and FaDu in their fractions of apoptosis cells. At 24 h, 48 h, or 72 h after irradiation, the fraction of apoptosis cells in RS-FaDu cells was lower than that in FaDu cells. All experiments were performed in triplicate wells; points, mean; bars, SD. **P < 0.01.

Radioresistance was measured by clonogenic survival assay following exposure to a range of radiation doses (0–6 Gy). As shown in Figure 1A, RS-FaDu and FaDu cells showed no difference in clonogenic formation ability when the radiation dose was 0 Gy. However, the RS-FaDu cells had significantly more and larger surviving colonies than did the control FaDu cells when the radiation dose was 4 Gy or 6 Gy. Clonogenic survival curves showed the surviving colony numbers of FaDu cells were significantly lower than those of RS-FaDu cells (**P < 0.01, both) at 4 Gy and 6 Gy (Figure 1B).

To further verify the radioresistant phenotype of RS-FaDu, RS-FaDu and FaDu cells were also examined by apoptosis assays. RS-FaDu and FaDu cells were treated with 4 Gy. Their fractions of apoptosis cells did not differ at 0 h, but at 24, 48, or 72 h after irradiation, the fractions of apoptotic RS-FaDu cells were much lower than those of FaDu cells (**P < 0.01, Figure 1C, 1D). These results indicated that RS-FaDu cells were much more radioresistant than their parent FaDu cells. These data indicated that the RS-FaDu subclone cell line was successfully established.

### LncRNA and mRNA profiles

**Figure 2:**
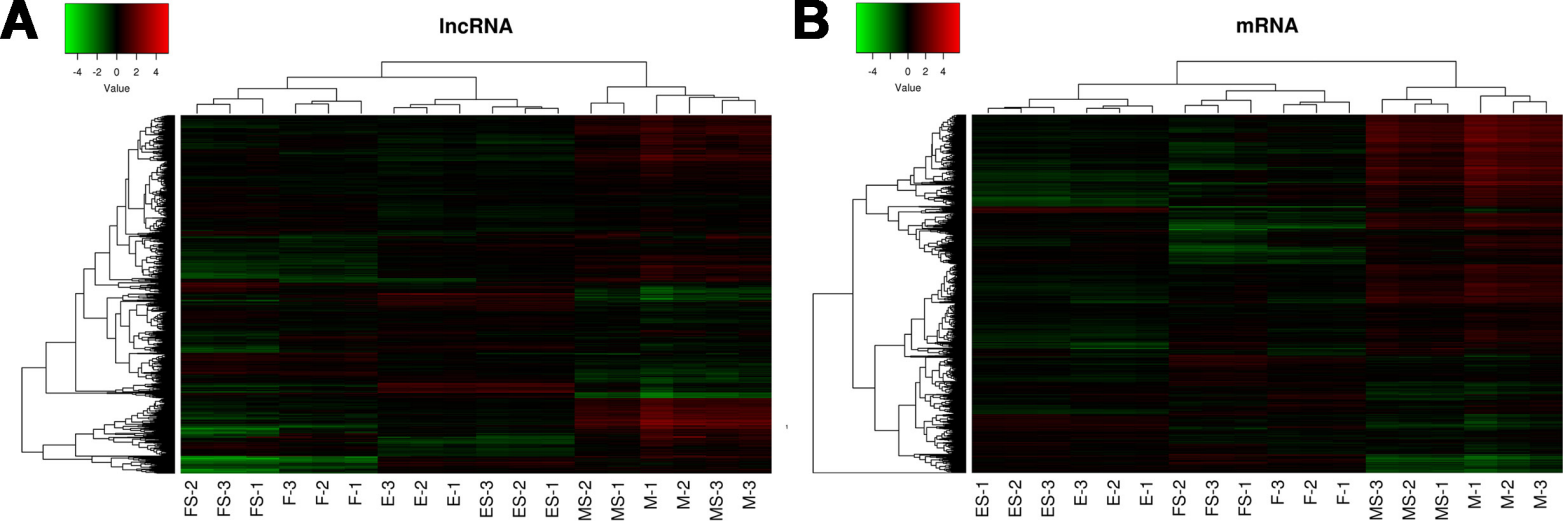
LncRNA (**A**) and mRNA (**B**) expression profiling by human lncRNA microarray. The sample tree on the top of the figure shows sample group information, which reflects relationships among samples. In the dendrogram, red indicates high relative expression, and blue indicates low relative expression. M, E, and F refer to 0 hr, 2 hrs, and 48 hrs after exposure to 4 Gy irradiation, respectively. The Arabic numerals presented the experiment repeats.

Hierarchical clustering is an unsupervised classification method that can separate multiple groups without the use of the group information. In microarray data analysis, cluster analysis grouped samples together based on expression intensity revealed differences between clustering group and true group results for removal of outlier samples. The dendrogram showed the relationships among lncRNA (Figure 2A) and mRNA (Figure 2B) expression patterns between RS-FaDu cells and FaDu cells at 0, 2, and 48 h, respectively, after 4 Gy radiation exposure.

**Figure 3:**
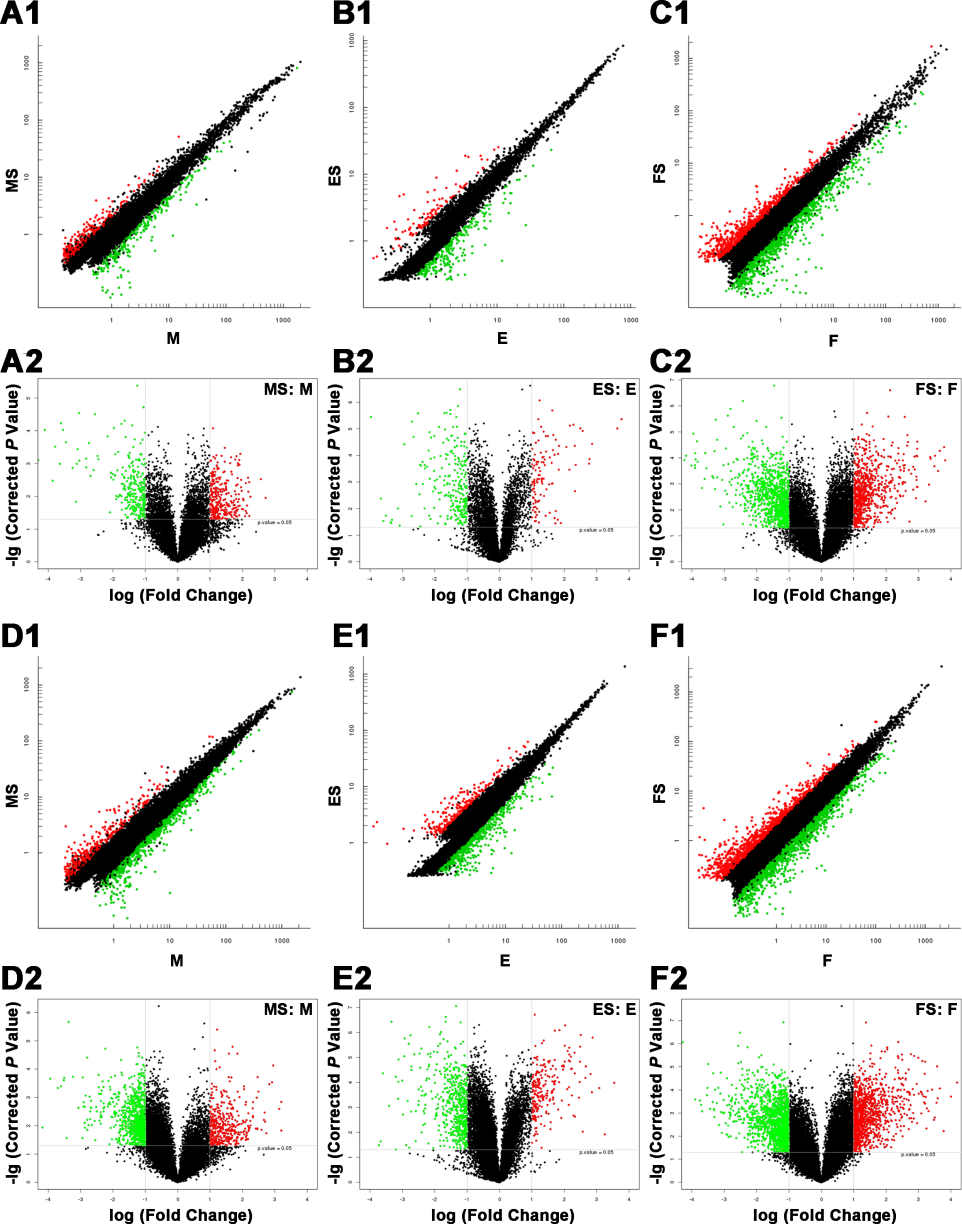
Differences in lncRNA and mRNA expression profiles between RS-FaDu cells and parental FaDu cells (**A1**–**F1**) Scatter plots. Scatter plot showed the differentially expressed lncRNAs (A1–C1) and mRNAs (D1–F1). The values on the X and Y axes from the averaged normalized signal values of groups of samples (log2 scaled). X-axis for control group (FaDu), and Y-axis for case group (RS-FaDu). All the lncRNAs/mRNAs without differential expression around the line Y = X in black. Points above Y = X and apart from Y = X are upregulated lncRNAs/mRNAs in red, and points below Y = X and apart from Y = X are downregulated lncRNAs/mRNAs in green, respectively. The threshold for differentially expressed genes was set at FC ≥ 2.0. (**A2**–**F2**) Volcano plots for the differences in lncRNA (A2–C2) and mRNA (D2–F2) expression between the case group (RS-FaDu) and control group (FaDu). The X-axis in the volcano plot represents FC (after log2 transformation) and the Y-axis in the plot represents *P*-value (after log transformation). The lncRNAs/mRNAs on the top left show downregulated lncRNAs/mRNAs (FC ≥ 2.0, *P* < 0.05). The lncRNAs/mRNAs on the top right show upregulated lncRNAs/mRNAs (FC ≥ 2.0, *P* < 0.05). Upregulated lncRNAs/mRNAs and downregulated lncRNAs/mRNAs are shown in red and green, respectively. MS vs. M, 0 h; ES vs. E, 2 h; FS vs. F, 48 h.

A scatter plot is a visualization method to show the differentially expressed lncRNAs (Figure 3, A1–C1) and mRNAs (Figure 3, D1–F1). The values plotted on the X and Y axes were the averaged normalized signal values of groups of samples (log2 scaled). The X-axis represents the control group (FaDu), while the Y-axis represents the case group (RS-FaDu). All lncRNAs/mRNAs that were not differentially expressed were around the line Y = X and labelled black. Points that were above and apart from Y = X were upregulated lncRNAs/mRNAs and labelled red, and points that were below and apart from Y = X were downregulated lncRNAs/mRNAs and labelled green, respectively. A volcano plot is another visualization method to show differences in lncRNA (Figure 3, A2–C2) and mRNA (Figure 3, D2–F2) expression between the case group (RS-FaDu) and the control group (FaDu). The X-axis in the volcano plot represents FC (after log2 transformation) and the Y-axis represents *P*-value (after log transformation). The lncRNAs/mRNAs on the top left were downregulated lncRNAs/mRNAs (FC ≥ 2.0, *P* value < 0.05). The lncRNAs/mRNAs on the top right were upregulated lncRNAs/mRNAs (FC ≥ 2.0, *P* value < 0.05). Upregulated lncRNAs/mRNAs and downregulated lncRNAs/mRNAs were labelled red and green, respectively. After the RS-FaDu and FaDu cells were treated with 4 Gy of irradiation, their lncRNA expression levels differed significantly in 575, 361, and 1714 lncRNAs (data not shown) at 0, 2, and 48 h, respectively. Of those, 302 were upregulated and 273 were downregulated at 0 h; 113 were upregulated and 248 were downregulated at 2 h; and 759 were upregulated and 955 were downregulated at 48 h, respectively. In addition, we identified 1249, 781, and 2521 (data not shown) mRNAs that were significantly differentially expressed at 0, 2, and 48 h, respectively. Of those, 387 were upregulated and 862 were downregulated at 0 h; 227 were upregulated and 554 were downregulated at 2 h; and 1089 were upregulated and 1432 were downregulated at 48 h. Volcano plot filtering was also used to identify the 10 most upregulated and downregulated lncRNAs (Table 1) and mRNAs (Table 2) in RS-FaDu cells at 0, 2, and 48 h after 4 Gy radiation, respectively.

**Table 1:** Ten most upregulated and downregulated lncRNAs in RS-FaDu cells at 0, 2, and 48 h after 4 Gy radiation

Probe name	FC (abs)	Regulation	lncRNA ID	Chr	Strand	Gene	Class	Time
p7538	6.58	up	ENST00000587434.1	17	+	ENSG00000267601.1	Antisense	0 h
p28462	5.94	up	ASO3704			ASO3704	Intergenic	0 h
p8064	4.75	up	ENST00000563172.1	18	+	ENSG00000261780.2	Intergenic	0 h
p19771	4.63	up	TCONS_00023442	15	+	XLOC_011287	Intergenic	0 h
p37817_v4	4.62	up	ENST00000607175.1	6	−	ENSG00000272468.1		0 h
p35075_v4	4.54	up	ENST00000594101.1	3	+	ENSG00000242086.3	Intergenic	0 h
p35072_v4	4.51	up	ENST00000597871.1	3	+	ENSG00000242086.3	Intergenic	0 h
p11909	4.41	up	ENST00000519700.1	3	+	ENSG00000242770.2	Intergenic	0 h
p6707	4.38	up	ENST00000578710.1	17	−	ENSG00000264673.1	Intergenic	0 h
p35069_v4	4.30	up	ENST00000438608.1	3	+	ENSG00000242086.3	Intergenic	0 h
p33918	19.81	down	hox-HOXD10-35	2	+	hox-HOXD10-35	Intronic	0 h
p33919	17.23	down	hox-HOXD10-36	2	+	hox-HOXD10-36	Intronic	0 h
p28077	14.53	down	nc-HOXD10-9	2	+	nc-HOXD10-9	Intronic	0 h
p5033	13.80	down	ENST00000556653.1	14	+	ENSG00000258914.1	Intergenic	0 h
p28072	12.49	down	nc-HOXD10-13	2	+	nc-HOXD10-13	Intronic	0 h
p10912	12.20	down	ENST00000430181.1	21	+	ENSG00000235890.1	Intronic	0 h
p28071	11.64	down	nc-HOXD10-12	2	+	nc-HOXD10-12	Intronic	0 h
p6908	10.93	down	ENST00000433510.1	17	−	ENSG00000233283.2	Intergenic	0 h
p8814	9.47	down	ENST00000601506.1	19	+	ENSG00000269495.1	Antisense	0 h
p33495	8.82	down	ENST00000589927.1	19	+	ENSG00000186526.7	Antisense	0 h
p29588	13.61	up	TCONS_00018436	10	−	XLOC_008730	Intergenic	2 h
p29587	12.47	up	TCONS_00017927	10	−	XLOC_008730	Intergenic	2 h
p22664	7.26	up	TCONS_00010875	5	−	XLOC_004700	Intergenic	2 h
p5789	6.87	up	ENST00000567091.1	16	−	ENSG00000260394.2	Divergent	2 h
p3381	6.81	up	ENST00000547963.1	12	−	ENSG00000249550.2	Intergenic	2 h
p3379	5.93	up	ENST00000550905.1	12	−	ENSG00000249550.2	Intergenic	2 h
p5993	5.64	up	ENST00000567668.1	16	−	ENSG00000260609.1	Intergenic	2 h
p19771	5.11	up	TCONS_00023442	15	+	XLOC_011287	Intergenic	2 h
p737	5.03	up	ENST00000453572.1	1	−	ENSG00000232184.1	Intronic	2 h
p22663	5.01	up	TCONS_00010233	5	−	XLOC_004700	Intergenic	2 h
p8814	23.31	down	ENST00000601506.1	19	+	ENSG00000269495.1	Antisense	2 h
p8817	15.69	down	ENST00000596286.1	19	+	ENSG00000268739.1	Antisense	2 h
p28076	12.71	down	nc-HOXD10-8	2	+	nc-HOXD10-8	Antisense	2 h
p33919	12.04	down	hox-HOXD10-36	2	+	hox-HOXD10-36	Intronic	2 h
p33918	11.42	down	hox-HOXD10-35	2	+	hox-HOXD10-35	Intronic	2 h
p28072	10.30	down	nc-HOXD10-13	2	+	nc-HOXD10-13	Intronic	2 h
p33495	10.15	down	ENST00000589927.1	19	+	ENSG00000186526.7	Antisense	2 h
p28071	10.01	down	nc-HOXD10-12	2	+	nc-HOXD10-12	Intronic	2 h
p8609	8.80	down	ENST00000595892.1	19	+	ENSG00000269640.1	Divergent	2 h
p28077	8.48	down	nc-HOXD10-9	2	+	nc-HOXD10-9	Intronic	2 h
p40301_v4	21.79	up	XR_427456.1	3	+			48 h
p3438	14.16	up	ENST00000545853.1	12	−	ENSG00000256732.1	Intergenic	48 h
p18725	13.84	up	TCONS_00020973	12	−	XLOC_010243	Intergenic	48 h
p33351	12.63	up	ENST00000420462.1	1	−	ENSG00000242663.1	Antisense	48 h
p11893	12.18	up	ENST00000462011.1	3	+	ENSG00000244464.1	Intergenic	48 h
p37817_v4	11.39	up	ENST00000607175.1	6	−	ENSG00000272468.1		48 h
p26490	11.25	up	uc004aej.3	9	−	BC065763	Intergenic	48 h
p26072	10.39	up	uc002oet.3	19	+	BC024306	Intergenic	48 h
p29587	10.28	up	TCONS_00017927	10	−	XLOC_008730	Intergenic	48 h
p14418	10.03	up	ENST00000584911.1	6	−	ENSG00000223414.2	Intergenic	48 h
p28077	88.86	down	nc-HOXD10-9	2	+	nc-HOXD10-9	Intronic	48 h
p33919	69.03	down	hox-HOXD10-36	2	+	hox-HOXD10-36	Intronic	48 h
p8814	52.88	down	ENST00000601506.1	19	+	ENSG00000269495.1	Antisense	48 h
p28072	49.57	down	nc-HOXD10-13	2	+	nc-HOXD10-13	Intronic	48 h
p28076	45.45	down	nc-HOXD10-8	2	+	nc-HOXD10-8	Antisense	48 h
p28071	41.28	down	nc-HOXD10-12	2	+	nc-HOXD10-12	Intronic	48 h
p33495	38.99	down	ENST00000589927.1	19	+	ENSG00000186526.7	Antisense	48 h
p33920	33.10	down	hox-HOXD11-34	2	+	hox-HOXD11-34	Intronic	48 h
p25262	26.48	down	XR_108533.1	3	−	LOC100505902	Intergenic	48 h
p33918	23.62	down	hox-HOXD10-35	2	+	hox-HOXD10-35	Intronic	48 h

**Table 2:** Ten most upregulated and downregulated mRNAs in RS-FaDu cells at 0, 2 and 48 h after 4 Gy radiation

Probe name	FC (abs)	Regulation	Genbank accession	Gene symbol	Time
A_21_P0005630	23.64873	up	NR_121672	LINC00824	0 h
A_33_P3258346	9.20869	up	NM_017523	XAF1	0 h
A_33_P3384287	9.20060	up	NM_002579	PALM	0 h
A_33_P3238533	8.80111	up	NM_001105528	CCDC178	0 h
A_23_P87013	7.73073	up	NM_001001522	TAGLN	0 h
A_33_P3381948	7.55051	up	NM_001080436	WTIP	0 h
A_23_P1029	7.11102	up	NM_017459	MFAP2	0 h
A_33_P3640690	6.67747	up	NM_001128128	ZEB1	0 h
A_24_P557479	6.51224	up	NM_017523	XAF1	0 h
A_33_P3237552	6.43079	up	NM_032843	FIBCD1	0 h
A_33_P3290780	52.36956	down	NM_001185156	IL24	0 h
A_33_P3260654	26.23980	down	EU030678		0 h
A_24_P684183	18.09698	down	NM_025257	SLC44A4	0 h
A_23_P304897	15.30950	down	NM_000623	BDKRB2	0 h
A_23_P128744	13.02192	down	NM_000710	BDKRB1	0 h
A_23_P122937	12.17637	down	NM_014800	ELMO1	0 h
A_21_P0009192	11.46747	down			0 h
A_23_P65189	11.36208	down	NM_000209	PDX1	0 h
A_23_P39315	10.78665	down	NM_021187	CYP4F11	0 h
A_23_P404494	10.34955	down	NM_002185	IL7R	0 h
A_21_P0005630	73.61931	up	NR_121672	LINC00824	2 h
A_33_P3238533	61.95869	up	NM_001105528	CCDC178	2 h
A_23_P69030	11.65424	up	NM_001850	COL8A1	2 h
A_33_P3245439	9.52713	up	NM_001250	CD40	2 h
A_23_P209055	8.46069	up	NM_001771	CD22	2 h
A_33_P3293675	7.34682	up	NM_006598	SLC12A7	2 h
A_24_P917886	7.22717	up	XM_006709947	MUC5AC	2 h
A_33_P3382177	7.02720	up	NM_003255	TIMP2	2 h
A_32_P530933	6.34961	up	NM_015617	PYGO1	2 h
A_23_P159721	6.29696	up	NM_004224	GPR50	2 h
A_33_P3393971	27.56643	down	NM_000299	PKP1	2 h
A_23_P23296	24.91828	down	NM_000299	PKP1	2 h
A_23_P143029	21.06592	down	NM_021192	HOXD11	2 h
A_24_P245379	13.24701	down	NM_002575	SERPINB2	2 h
A_33_P3220911	12.74436	down	NM_004335	BST2	2 h
A_33_P3226810	12.12693	down	NM_003810	TNFSF10	2 h
A_23_P404494	10.63455	down	NM_002185	IL7R	2 h
A_24_P236935	10.10874	down	NM_001012964	KLK6	2 h
A_21_P0011633	9.24221	down	NM_000526	KRT14	2 h
A_23_P39315	9.24213	down	NM_021187	CYP4F11	2 h
A_33_P3238533	132.03939	up	NM_001105528	CCDC178	48 h
A_21_P0005630	41.69042	up	NR_121672	LINC00824	48 h
A_23_P161190	30.52496	up	NM_003380	VIM	48 h
A_23_P70468	29.94054	up	NM_012367	OR2B6	48 h
A_33_P3340014	18.30612	up	NM_016157	TRO	48 h
A_33_P3514487	16.05847	up	NM_198481	VSTM1	48 h
A_24_P211849	13.71514	up	NM_001166220	TBX20	48 h
A_23_P421379	12.63051	up	NM_000612	IGF2	48 h
A_23_P69030	12.08606	up	NM_001850	COL8A1	48 h
A_23_P41804	11.93085	up	NM_033120	NKD2	48 h
A_23_P39315	39.50620	down	NM_021187	CYP4F11	48 h
A_23_P143029	38.05859	down	NM_021192	HOXD11	48 h
A_23_P50710	31.23669	down	NM_001082	CYP4F2	48 h
A_33_P3393971	26.30644	down	NM_000299	PKP1	48 h
A_23_P23296	24.31587	down	NM_000299	PKP1	48 h
A_24_P42693	23.02732	down	NM_021187	CYP4F11	48 h
A_23_P108280	20.67472	down	NM_023944	CYP4F12	48 h
A_24_P684183	19.34901	down	NM_025257	SLC44A4	48 h
A_23_P138541	14.88679	down	NM_003739	AKR1C3	48 h
A_23_P300781	13.48931	down	NM_013316	CNOT4	48 h

**Figure 4:**
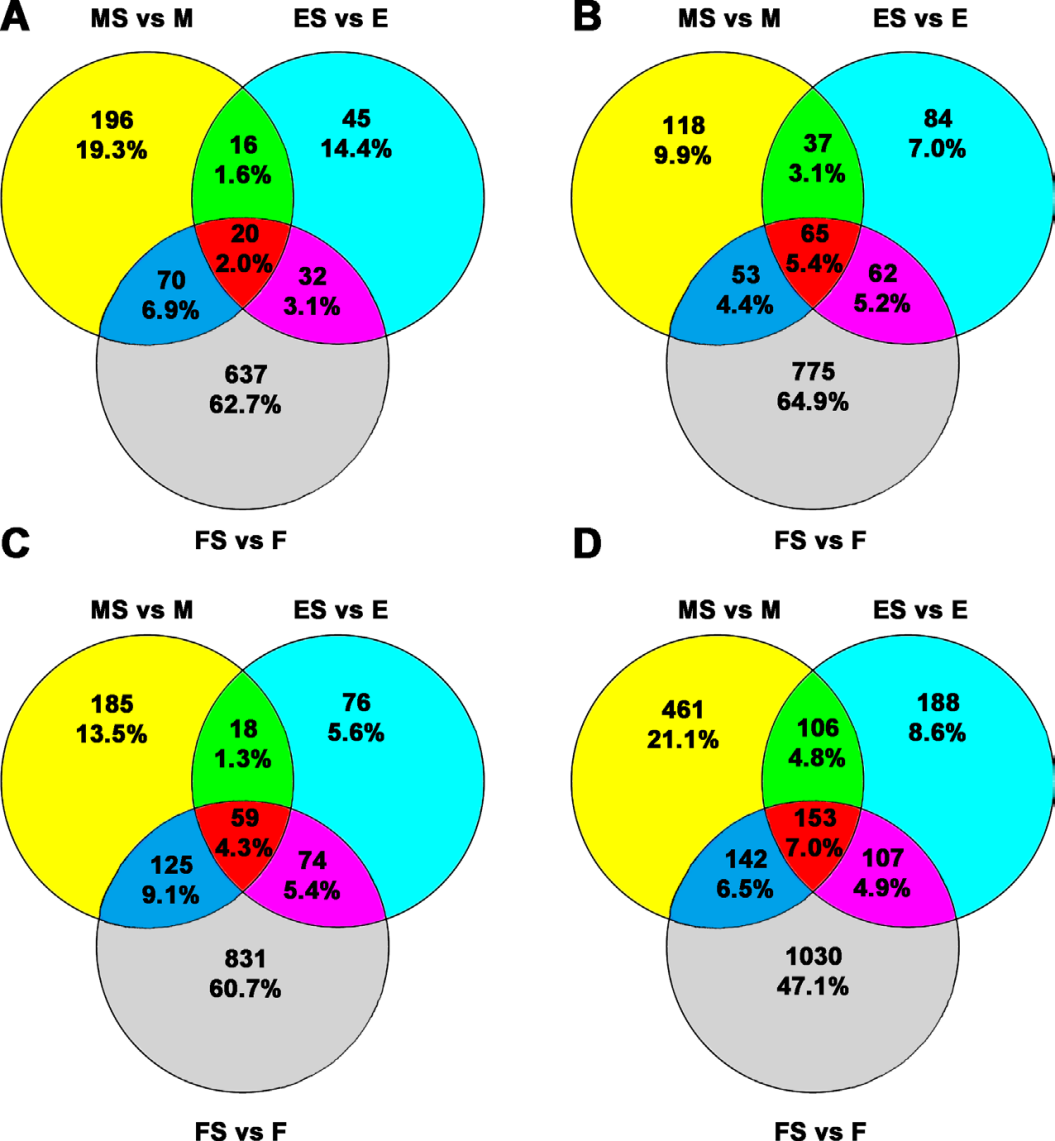
Venn diagram for the common and exclusively expressed lncRNAs and mRNAs from each group FaDu and RS-FaDu cells at 0, 2, and 48 h after irradiation with 4 Gy. Different lncRNAs and mRNAs between FaDu and RS-FaDu as determined by microarray analysis for overlapping signature. (**A**) The overlapping results of upregulated differentially expressed lncRNAs. (**B**) The overlapping results of downregulated differentially expressed lncRNAs. (**C**) The overlapping results of upregulated differentially expressed mRNAs. (**D**) The overlapping results of downregulated differentially expressed mRNAs. MS vs. M, 0 h; ES vs. E, 2 h; FS vs. F, 48 h.

Venn diagrams of the numbers and percentages of differentially expressed genes are shown in Figure 4. The results showed that there were 20 (2.0%) common differentially upregulated lncRNAs (Figure 4A) and 65 (5.4%) common differentially downregulated lncRNAs (Figure 4B) for the three groups (Supplementary Table 1). In addition, the numbers of common upregulated (Figure 4C) and downregulated (Figure 4D) differentially expressed mRNAs were 59 (4.3%) and 153 (7.0%), respectively (Supplementary Table 2).

### Validation of differential lncRNA expression by qRT-PCR

**Figure 5:**
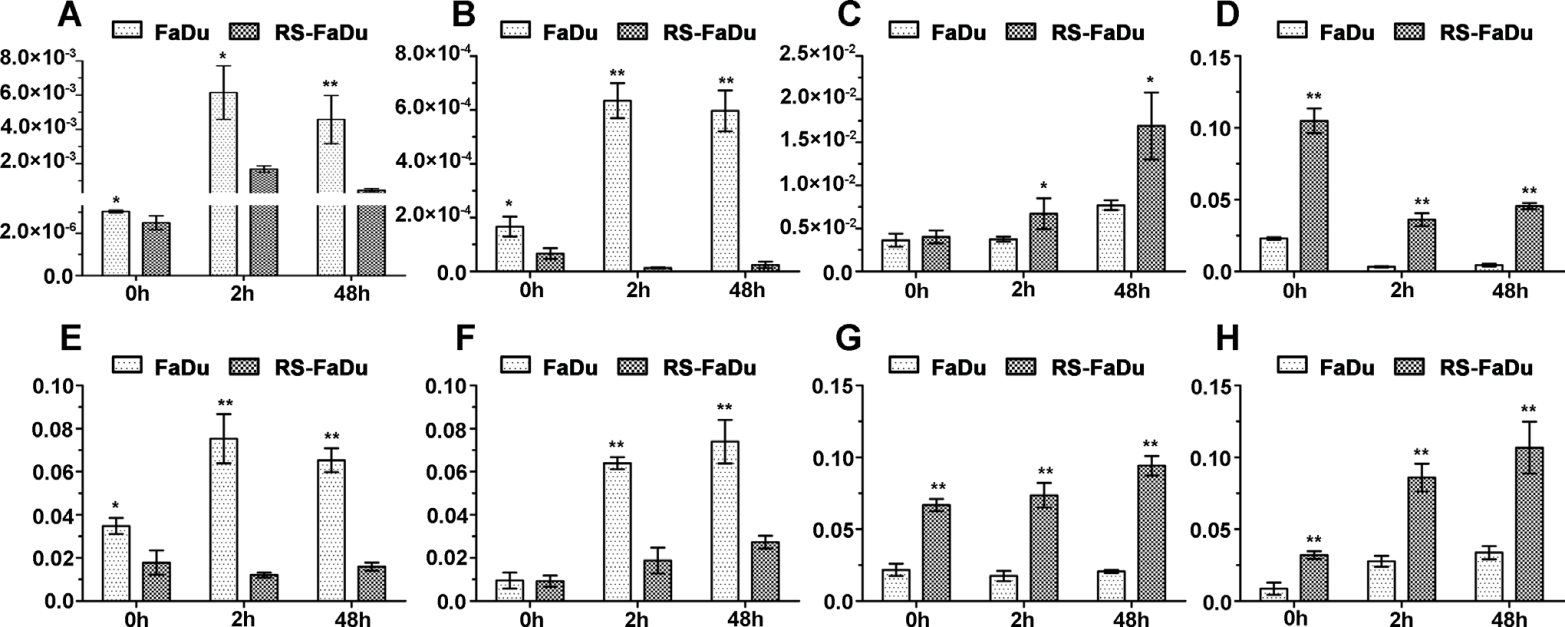
Validation of differential lncRNA expressions by qRT-PCR (**A**) ENST00000470135; (**B**) hox-HOXD10-35; (**C**) TCONS_00010875; (**D**) TCONS_00018436; (**E**) CKMT1A; (**F**) GPNMB; (**G**) FBLN5; (**H**) GDA. After normalization to ACTB, data were presented as mean ± SD. *n* = 3, *P < 0.05, **P < 0.01.

The qRT-PCR was used to confirm the reliability and validity of microarray data. We selected four lncRNAs (ENST00000470135, TCONS_00010875, TCONS_00018436, and hox-HOXD10-35) for validation since these lncRNAs had consistent up- or downregulations at the three time points and their FC values were prominent at some time points. Additionally, four mRNAs (CKMT1A, GPNMB, FBLN5, and GDA) were validated as well due likely to their potential roles in irradiation response or radioresistance. The relative expression levels of the target RNAs were given as ratios of β-actin transcript levels in the same RNA samples. As shown in Figure 5, the expression levels of these eight genes were consistent with the microarray results, indicating the reliability of the microarray data and correlation of these genes with radioresistance.

### Potential roles of TCONS_00018436 in regulation of radioresistance of HSCC

**Figure 6:**
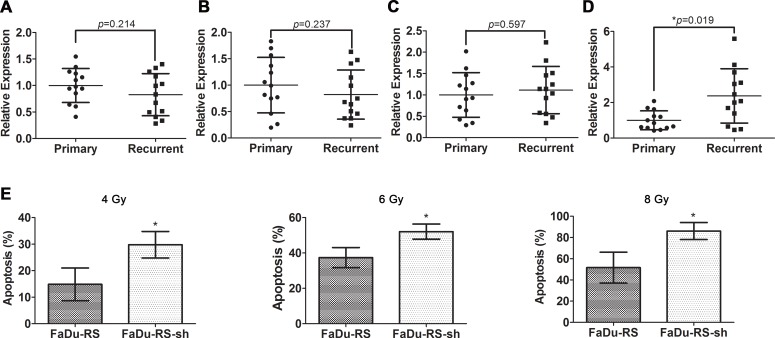
Potential roles of TCONS_00018436 in radioresistance of HSCC cells The relative expression of ENST00000470135 (**A**), hox-HOXD10-35 (**B**), TCONS_00010875 (**C**) and TCONS_00018436 (**D**) in primary *vs*. recurrent HSCC tissue samples were measured by qRT-PCR. Their expression in each sample was normalized to the mean expression of their respective primary samples. Data were presented as mean ± SD. *n* = 13, *P < 0.05. (**E**) FaDu-RS cells and those stably transfected with shRNA of TCONS_00018436 were both treated with 4 Gy, 6 Gy, and 8 Gy irradiation, respectively. The fractions of apoptotic cells were determined by using Annexin V/PI dual staining after 48 h. Data were presented as mean ± SD. *n* = 3, *P < 0.05.

We then assessed the expression of the four validated lncRNAs in primary tumor tissues of HSCC patients *versus* their recurrent ones after postoperative radiotherapy, using qRT-PCR. The expression of the three lncRNAs showed no remarkable difference between two groups of samples (Figure 6A–6C), while the significant upregulation of TCONS_00018436 in relapsed tumor samples was found (Figure 6D, *P < 0.05). Further, after we stably knocked down TCONS_00018436 in FaDu-RS cells using lentiviral transfection, both transfected FaDu-RS cells (FaDu-RS-sh) and FaDu-RS cells were treated with 4 Gy, 6 Gy and 8 Gy irradiation, respectively. The apoptotic cells at 48 h after irradiation were determined by Annexin V-FITC/PI and flow cytometry. As shown in Figure 6E, depletion of TCONS_00018436 significantly sensitized RS-FaDu cells to the indicated doses of radiation (*P < 0.05), indicating that upregulated TCONS_00018436 might control radioresistance of HSCC cells during exposure to radiation. However, the underlying mechanism remains to be further investigated.

### Bioinformatics analyses

In order to identify potential protein regulators involved in radioresistance of HSCC, we performed pathway enrichment analysis by functionally annotating and differentially expressed mRNAs in FaDu *vs*. FaDu-RS cells at 0, 2, and 48 h after irradiation. The detailed information on significantly enriched pathway terms and dysregulated mRNAs at the three time points involved in them was presented in Supplementary Tables 3, 4, 5, respectively. The most significant 30 terms sorted by corrected *P*-value were separately listed in three histograms, according to the time points after irradiation shown in Supplementary Figure 1. This analysis approach may help identify altered expression of mRNAs involved in pathways associated with radioresistance, whose dysregulation might have an impact on sensitivity of HSCC to radiation.

An increasing number of lncRNAs have been shown to regulate expression of target genes in *cis* or in *trans*. Thus, we found significant correlations between dysregulated lncRNAs and mRNAs at 0, 2, and 48 h after irradiation, respectively. After lncRNA and mRNA correlation, *cis*-prediction and *trans*-prediction were both applied to the data. The final lncRNA prediction results are the combination of results from these two prediction parts at 0, 2, and 48 h as shown in Tables 3, 4, 5, respectively. These pathway enrichment analysis may provide some mRNA candidates that are potentially associated with radioresistance. Furthermore, we might further identify the lncRNAs in regulating the expressions of nearby or distant genes which encode these mRNAs based on the prediction results.

**Table 3:** lncRNA target prediction of RS-FaDu vs. FaDu at 0 h

lncRNA	mRNA	Correlation	*P*-value	Direction (lncRNA-mRNA)	cisregulation	transregulation
p3758	A_33_P3421178	0.999026552	0.000001421	down-down	Sense	
p13056	A_21_P0012973	0.993822774	0.000057119	up-up	Sense	
p25219	A_33_P3368495	0.998540055	0.000003196	down-down		miRNA sequestration
p7538	A_33_P3382177	0.999529708	0.000000332	up-up	Antisense	
p35072_v4	A_19_P00315941	0.997663892	0.000008180	up-up	Sense	
p12195	A_19_P00316857	0.994153893	0.000051166	up-up	Sense	
p26166	A_24_P557479	−0.997108515	0.000012529	down-up		miRNA sequestration
p29619	A_21_P0007233	0.992506995	0.000084007	down-down	Sense	
p38695_v4	A_23_P148919	−0.99696864	0.000013770	up-down		miRNA sequestration
p192	A_23_P148919	0.994529457	0.000044808	down-down	Antisense	
p1665	A_33_P3278211	0.994573678	0.000044088	down-down	Sense	
p30074	A_33_P3289416	0.990995817	0.000121248	down-down		miRNA sequestration
p10587	A_24_P115199	−0.99188824	0.000098434	up-down		miRNA sequestration
p8323	A_24_P115199	−0.997557313	0.000008943	up-down		miRNA sequestration
p687	A_21_P0001239	0.991689858	0.000103301	down-down	Sense	
p686	A_21_P0001239	0.999058836	0.000001328	down-down	Sense	
p34021_v4	A_21_P0001239	0.998331716	0.000004172	down-down	Sense	
p7388	A_33_P3341836	0.997644418	0.000008317	up-up		miRNA sequestration
p687	A_32_P212373	0.991025177	0.000120460	down-down	Sense	
p686	A_32_P212373	0.998954092	0.000001640	down-down	Sense	
p34021_v4	A_32_P212373	0.998577934	0.000003032	down-down	Sense	
p686	A_21_P0001238	0.994752897	0.000041226	down-down	Sense	
p34021_v4	A_21_P0001238	0.992305807	0.000088573	down-down	Sense	
p38695_v4	A_33_P3414487	−0.99790579	0.000006574	up-down		miRNA sequestration
p10433	A_23_P210425	0.993170163	0.000069811	up-up	Antisense	
p4963	A_23_P304897	0.994892042	0.000039070	down-down	Intronic	
p6222	A_24_P80135	0.997657113	0.000008227	down-down		miRNA sequestration
p15211	A_23_P320878	0.993544033	0.000062385	down-down		miRNA sequestration

**Table 4:** LncRNA target prediction of RS-FaDu vs. FaDu at 2 h

lncRNA	mRNA	Correlation	*P*-value	Direction (lncRNA-mRNA)	cisregulation	transregulation
p20305	A_33_P3266898	−0.996352571	0.000019931	up-down		miRNA sequestration
p30194	A_33_P3266898	−0.996726605	0.000016055	up-down		miRNA sequestration
p33576	A_32_P131031	0.992702048	0.000079696	down-down	Intergenic (10 k)	
p36121_v4	A_21_P0004245	0.990861638	0.000124883	down-down	Sense	
p8814	A_24_P236935	0.995770285	0.000026798	down-down	Antisense	
p687	A_24_P915692	0.990515655	0.000134503	down-down		miRNA sequestration
p24838	A_33_P3278211	0.998051663	0.000005690	down-down	Sense	
p1665	A_33_P3278211	0.998814061	0.000002109	down-down	Sense	
p37870_v4	A_33_P3289416	−0.990915820	0.000123409	up-down		miRNA sequestration
p25347	A_24_P940149	−0.996508538	0.000018264	up-down		miRNA sequestration
p25347	A_32_P152437	0.996042331	0.000023464	up-up		miRNA sequestration
p30340	A_24_P602871	0.995141025	0.000035357	down-down		miRNA sequestration
p25605	A_23_P329112	0.996929698	0.000014126	up-down		miRNA sequestration
p25347	A_23_P329112	0.994658656	0.000042719	up-down		miRNA sequestration
p11034	A_24_P66027	0.991916997	0.000097738	down-down	Antisense	
p28133	A_24_P115199	0.997640377	0.000008345	down-down		miRNA sequestration
p30155	A_24_P115199	0.990576762	0.000132778	down-down		miRNA sequestration
p33798	A_23_P60339	−0.994723656	0.000041686	up-down		miRNA sequestration
p687	A_32_P212373	0.992913328	0.000075153	down-down	Sense	
p686	A_32_P212373	0.994692846	0.000042174	down-down	Sense	
p34021_v4	A_32_P212373	0.991057378	0.000119598	down-down	Sense	
p20305	A_23_P208389	0.996581866	0.000017505	up-up		miRNA sequestration
p34907_v4	A_33_P3333554	0.990605549	0.000131969	down-down		miRNA sequestration
p10433	A_23_P210425	0.998311004	0.000004277	up-up	Antisense	
p30335	A_23_P157022	0.991277419	0.000113793	down-down		miRNA sequestration
p15938	A_21_P0005906	0.995291866	0.000033198	down-down	Intergenic (10 k)	
p34907_v4	A_33_P3378430	0.991616881	0.000105120	down-down		miRNA sequestration
p20305	A_32_P31618	−0.992612720	0.000081656	up-down		miRNA sequestration
p15519	A_33_P3233273	0.990760089	0.000127670	down-down	Intergenic (10 k)	

**Table 5:** LncRNA target prediction of RS-FaDu vs. FaDu at 48 h

lncRNA	mRNA	Correlation	*P*-value	Direction (lncRNA-mRNA)	cisregulation	transregulation
p24892	A_33_P3250133	0.993774158	0.000058021	up-up		miRNA sequestration
p36462_v4	A_24_P111242	−0.997243502	0.000011387	down-up		miRNA sequestration
p2627	A_21_P0014268	0.996216336	0.000021447	up-up	Sense	
p25293	A_23_P38813	−0.997569809	0.000008852	up-down		miRNA sequestration
p24618	A_23_P38813	0.996136217	0.000022364	down-down		miRNA sequestration
p37916_v4	A_23_P38813	−0.991608950	0.000105319	up-down		miRNA sequestration
p44617_v4	A_23_P38813	−0.993806999	0.000057411	up-down		miRNA sequestration
p25051	A_23_P38813	−0.995045168	0.000036765	up-down		miRNA sequestration
p28498	A_23_P38813	−0.994453852	0.000046054	up-down		miRNA sequestration
p122	A_23_P38813	−0.994491445	0.000045433	up-down		miRNA sequestration
p3143	A_23_P38813	0.997519222	0.000009224	down-down		miRNA sequestration
p11437	A_23_P38813	−0.995453161	0.000030964	up-down		miRNA sequestration
p37026_v4	A_23_P38813	−0.994529667	0.000044805	up-down		miRNA sequestration
p23798	A_23_P38813	0.992085159	0.000093719	down-down		miRNA sequestration
p29606	A_23_P38813	−0.998223527	0.000004731	up-down		miRNA sequestration
p28658	A_23_P38813	−0.993772836	0.000058046	up-down		miRNA sequestration
p24771	A_21_P0014060	0.995431495	0.000031259	up-up	Sense	
p34907_v4	A_24_P319364	−0.996770625	0.000015626	down-up		miRNA sequestration
p29827	A_33_P3336622	0.994560238	0.000044306	up-up		miRNA sequestration
p29606	A_33_P3336622	0.993785701	0.000057806	up-up		miRNA sequestration
p26063	A_33_P3390723	0.990423713	0.000137119	up-up		miRNA sequestration
p24892	A_23_P45799	−0.997314248	0.000010810	up-down		miRNA sequestration
p34907_v4	A_24_P129232	−0.992791851	0.000077749	down-up		miRNA sequestration
p18271	A_24_P684183	−0.990582868	0.000132606	up-down		miRNA sequestration
p44617_v4	A_24_P684183	−0.990879668	0.000124391	up-down		miRNA sequestration
p18272	A_24_P684183	−0.993001983	0.000073287	up-down		miRNA sequestration
p8313	A_23_P165186	0.990188970	0.000143912	up-up	Bidirectional	
p7538	A_33_P3382177	0.990676885	0.000129976	up-up	Antisense	
p41822_v4	A_32_P126698	0.993882119	0.000056028	up-up		miRNA sequestration
p25293	A_23_P149099	−0.991073247	0.000119175	up-down		miRNA sequestration
p18271	A_23_P149099	−0.991096396	0.000118558	up-down		miRNA sequestration
p823	A_23_P149099	−0.992591892	0.000082117	up-down		miRNA sequestration
p23798	A_23_P149099	0.993960353	0.000054606	down-down		miRNA sequestration
p25999	A_23_P149099	−0.991209023	0.000115582	up-down		miRNA sequestration
p25051	A_24_P382630	−0.998198512	0.000004865	up-down		miRNA sequestration
p28498	A_24_P382630	−0.991372680	0.000111325	up-down		miRNA sequestration
p16320	A_23_P62679	−0.995317469	0.000032838	up-down		miRNA sequestration
p13683	A_33_P3233841	0.993630385	0.000060729	up-up	Antisense	
p21313	A_19_P00317856	0.993327189	0.000066641	up-up	Sense	miRNA sequestration
p36492_v4	A_33_P3221748	0.995546108	0.000029712	up-up	Sense	
p19344	A_24_P250535	0.991880869	0.000098613	up-up		miRNA sequestration
p8693	A_24_P250535	0.991653359	0.000104209	up-up		miRNA sequestration
p14566	A_19_P00319254	0.991633933	0.000104694	down-down	Sense	
p22664	A_23_P41804	0.995620949	0.000028722	up-up	Intergenic (10 k)	
p22663	A_23_P41804	0.997949994	0.000006299	up-up	Intergenic (10 k)	
p37916_v4	A_33_P3215277	0.992325400	0.000088123	up-up		miRNA sequestration
p28498	A_33_P3215277	0.993349818	0.000066190	up-up		miRNA sequestration
p20125	A_33_P3256490	0.993114016	0.000070962	up-up		miRNA sequestration
p2107	A_33_P3256490	0.996526411	0.000018078	up-up		miRNA sequestration
p33596	A_23_P347468	0.990934613	0.000122899	down-down		miRNA sequestration
p4313	A_33_P3239084	0.991022154	0.000120541	down-down		miRNA sequestration
p26099	A_23_P381368	0.992698802	0.000079767	down-down	Intergenic (10 k)	
p28109	A_23_P381368	0.998436798	0.000003663	down-down		miRNA sequestration
p5755	A_24_P56894	0.990801171	0.000126538	up-up		miRNA sequestration
p16320	A_24_P56894	0.991795931	0.000100684	up-up		miRNA sequestration
p2195	A_23_P134935	0.995584780	0.000029198	up-up		miRNA sequestration
p18273	A_23_P76749	0.991517681	0.000107619	up-up		miRNA sequestration
p18273	A_23_P336929	0.990945545	0.000122604	up-up		miRNA sequestration
p3234	A_24_P380022	−0.990116941	0.000146030	up-down		miRNA sequestration
p16320	A_33_P3319593	−0.991594493	0.000105682	up-down		miRNA sequestration
p37870_v4	A_33_P3319593	−0.993768247	0.000058131	up-down		miRNA sequestration
p36411_v4	A_24_P941217	−0.992930705	0.000074786	down-up		miRNA sequestration
p25274	A_24_P941217	0.993605918	0.000061196	up-up		miRNA sequestration
p30340	A_23_P209320	−0.991624411	0.000104932	down-up		miRNA sequestration
p34907_v4	A_33_P3424297	0.991763098	0.000101490	down-down		miRNA sequestration
p30340	A_33_P3424297	0.990792516	0.000126776	down-down		miRNA sequestration
p8323	A_33_P3418394	0.996071022	0.000023125	up-up		miRNA sequestration
p11437	A_33_P3418394	0.992377207	0.000086939	up-up		miRNA sequestration
p1517	A_21_P0014248	0.993602194	0.000061267	up-up	Sense	
p14566	A_19_P00322225	0.991838124	0.000099652	down-down	Sense	
p26790	A_24_P940149	0.993717158	0.000059087	down-down	Antisense	
p17915	A_23_P416305	−0.993783331	0.000057850	down-up		miRNA sequestration
p750	A_21_P0010797	0.996064600	0.000023201	up-up	Sense	
p29253	A_32_P173058	0.992308714	0.000088506	up-up		miRNA sequestration
p36497_v4	A_19_P00319372	0.993618413	0.000060957	up-up	Intronic	
p36069_v4	A_19_P00319372	0.994980191	0.000037734	up-up	Sense	
p3143	A_23_P383031	0.994487956	0.000045490	down-down		miRNA sequestration
p18271	A_24_P409042	−0.993703568	0.000059343	up-down		miRNA sequestration
p24618	A_24_P409042	0.997488035	0.000009457	down-down		miRNA sequestration
p18273	A_24_P409042	−0.991108770	0.000118230	up-down		miRNA sequestration
p25051	A_24_P409042	−0.994189287	0.000050548	up-down		miRNA sequestration
p18272	A_24_P409042	−0.994084150	0.000052392	up-down		miRNA sequestration
p7539	A_24_P409042	−0.991072129	0.000119205	up-down		miRNA sequestration
p18273	A_23_P397856	−0.993936073	0.000055045	up-down		miRNA sequestration
p6823	A_24_P254346	−0.995095345	0.000036024	up-down		miRNA sequestration
p41766_v4	A_23_P97021	−0.991498540	0.000108105	up-down		miRNA sequestration
p29786	A_23_P97021	−0.995760159	0.000026926	up-down		miRNA sequestration
p9299	A_23_P97021	−0.990583844	0.000132579	up-down		miRNA sequestration
p28283	A_23_P97021	−0.991229915	0.000115034	up-down		miRNA sequestration
p18271	A_24_P373286	0.992099626	0.000093377	up-up		miRNA sequestration
p24976	A_23_P86182	0.994761294	0.000041094	down-down		miRNA sequestration
p16298	A_32_P90080	0.997392142	0.000010193	down-down		miRNA sequestration
p7391	A_23_P371885	0.995164968	0.000035010	up-up		miRNA sequestration
p16320	A_21_P0013080	−0.991724549	0.000102441	up-down		miRNA sequestration
p37870_v4	A_21_P0013080	−0.990354772	0.000139097	up-down		miRNA sequestration
p4547	A_23_P329112	−0.990672972	0.000130084	down-up		miRNA sequestration
p11094	A_23_P26704	−0.990068457	0.000147464	up-down		miRNA sequestration
p8313	A_23_P26704	−0.992380572	0.000086862	up-down		miRNA sequestration
p29606	A_23_P26704	−0.996163250	0.000022053	up-down		miRNA sequestration
p15693	A_23_P304682	−0.990624661	0.000131433	up-down		miRNA sequestration
p12560	A_23_P304682	−0.990915532	0.000123416	up-down		miRNA sequestration
p40979_v4	A_33_P3391005	−0.996196971	0.000021667	down-up		miRNA sequestration
p38890_v4	A_32_P831181	−0.996877479	0.000014610	up-down		miRNA sequestration
p3356	A_21_P0014571	0.991097745	0.000118522	up-up	Sense	
p12621	A_19_P00317034	0.993856369	0.000056500	down-down	Sense	
p5975	A_23_P63660	−0.991237992	0.000114823	up-down		miRNA sequestration
p9442	A_23_P63660	−0.993997587	0.000053935	up-down		miRNA sequestration
p122	A_21_P0001724	0.990393941	0.000137971	up-up	Sense	
p24892	A_23_P211748	0.992553680	0.000082965	up-up		miRNA sequestration
p1517	A_23_P161190	0.990268562	0.000141591	up-up	Intergenic (10 k)	
p29965	A_23_P208389	−0.999649344	0.000000184	down-up		miRNA sequestration
p20045	A_23_P208389	−0.993224778	0.000068700	down-up		miRNA sequestration
p33596	A_23_P146209	0.994585549	0.000043895	down-down		miRNA sequestration
p34907_v4	A_33_P3333554	0.992145626	0.000092295	down-down		miRNA sequestration
p38352_v4	A_23_P155666	0.997567735	0.000008867	down-down		miRNA sequestration
p36486_v4	A_23_P64792	−0.992286627	0.000089015	down-up		miRNA sequestration
p23949	A_23_P64792	0.992666927	0.000080464	up-up		miRNA sequestration
p25165	A_23_P50897	0.990700732	0.000129312	down-down	Antisense	
p10750	A_33_P3303305	0.991255110	0.000114375	down-down	Intronic	
p33919	A_23_P337201	0.997063796	0.000012919	down-down		miRNA sequestration
p1358	A_21_P0010738	0.996673835	0.000016577	down-down	Sense	
p6352	A_23_P163306	0.993659613	0.000060173	down-down		miRNA sequestration
p18271	A_24_P109652	0.992664902	0.000080508	up-up		miRNA sequestration
p2107	A_21_P0006705	0.998881367	0.000001876	up-up	Sense	
p4653	A_23_P304897	0.995441532	0.000031122	down-down	Intronic	
p4963	A_23_P304897	0.999067618	0.000001304	down-down	Intronic	
p39057_v4	A_33_P3259542	0.993876252	0.000056136	up-up		miRNA sequestration
p26034	A_33_P3259542	0.990129120	0.000145671	up-up		miRNA sequestration
p6898	A_21_P0014351	0.993996895	0.000053948	down-down	Sense	
p34009_v4	A_23_P104188	0.992257395	0.000089690	down-down	Intergenic (10 k)	
p34993_v4	A_23_P117582	0.992864673	0.000076188	up-up		miRNA sequestration
p25051	A_23_P117582	0.990949567	0.000122495	up-up		miRNA sequestration

## DISCUSSION

To investigate the role of lncRNAs in the radioresistance of HSCC, we first observed the expression profiles of lncRNA in our established radioresistant HSCC cell model, *i.e*. RS-FaDu. The expression levels of lncRNA in the RS-FaDu and the parental FaDu cells were determined by microarray analysis immediately, at 2 h or 48 h after exposure to 4 Gy irradiation. This approach enabled us to observe the time-course differential expression patterns of lncRNA and mRNA in RS-FaDu *vs*. parental FaDu cells at the early and late stages of their irradiation response. Extracellular stimulation can bring about a rapid change on transcription of related genes. However, it is hard to determine the exact time points of each stage. Borràs-Fresneda, et al. found that a greatly differential transcriptional response in the radioresistant cell line was induced at 4 h after irradiation compared with the radiosensitive one [27]. To make transcriptomic analyses of the radiation response in head and neck squamous cell carcinoma subclones with different radiosensitivity, Michna, et al. even detected gene expression at 0.25, 2, 7, 12, 24, 48, 72 and 96 h after irradiation by microarray [28]. According to their experience, we selected these time points to roughly observe the early and late transcriptional response of FaDu and FaDu-RS cells to irradiation in the current study. Additionally, Li et al also carried out research on the relationship between lncRNA and radioresistance in nasopharyngeal carcinoma through genome-wide analyses [29]. However, the authors did not look at response time, which might effectively narrow our search for target lncRNAs and mRNAs.

Furthermore, we identified lncRNAs and mRNAs that were up- or downregulated at above three time points, and they were considered more likely to be involved in HSCC radioresistance. Subsequent validation experiments not only confirmed the reliability of the microarray data but also provided four lncRNA or mRNA candidates for our future mechanism study. Among these, TCONS_00018436 was considered more promising, due to that its potential role was preliminarily verified by its upregulated expression in relapsed tumor samples posterior to radiotherapy and loss-of-function assays.

Considering that altered response processes might occur at different stages after irradiation, we functionally annotated dysregulated mRNAs and performed bioinformatics analyses according to the respective response times. Among the most significantly enriched pathway terms, several attracted our attention because of their close relationship with radioresistance, such as the p53 signaling pathway at 0 h and at 2 h [30, 31], and the Wnt signaling pathway at 48 h [32]. These results offer us preferential pathways in which to study the mechanisms underlying HSCC radioresistance. From these results, some candidate mRNAs could be identified based on their altered expression profiles of mRNAs as well.

LncRNAs are well known to affect the expression of target genes in *cis* or in *trans* through binding promoter regions of specific sequences, recruiting relevant transcription factors, and sequestering the interaction of miRNAs with target mRNAs, etc [33–36]. Thus, we identified dysregulated lncRNAs and their predicted mRNA targets on the basis of complementary base sequences and expression changes from our microarray data. According to dysregulation and association of mRNAs with radioresistance-related pathways in the pathway enrichment analysis, their matched lncRNAs could be found in our prediction results. We thought that it was likely a feasible way to search for lncRNA candidates for the further study.

Emerging evidence indicates that lncRNAs might function as competing endogenous RNAs by sponging miRNAs in a variety of cancers [37–40]. Notably, the lncRNAs NEAT1 [25] and MALAT1 [13, 24] have been shown to modulate radioresistance via sequestration of related miRNAs in nasopharyngeal carcinoma as well as high-risk human papillomavirus-positive cervical cancer. The regulatory roles of miRNAs are well established in the radioresistance of cancers [41–45], suggesting that we should explore the molecular mechanisms of lncRNAs in HSCC radioresistance from the perspective of lncRNA-miRNA-mRNA axes. Using sequence pairing, a number of dysregulated lncRNAs from our microarray data and matched potential miRNA targets were identified (data not shown) in preparation for verifying our hypothesis.

In addition to the exploration of mechanisms underlying HSCC radioresistance, the identification of biomarkers for predicting radioresistant HSCC is of great clinical significance. Recently, a growing number of circulating or tissue-derived lncRNAs have been shown to be correlated with clinicopathological characteristics in patients with cancer [46–48], making them promising candidate biomarkers of malignancy. Given the close relationship between lncRNAs and radioresistant HSCC cells discussed above, lncRNAs have the potential to become novel biomarkers for the evaluation of HSCC radioresistance. We further plan to measure the expression levels of candidate lncRNAs in tumor tissues as well as blood samples from HSCC patients and explore the correlation between expression levels and different responses of HSCC patients to routine radiation therapy.

In this study, we for the first time have comprehensively demonstrated the time-course expression profiles of human lncRNAs/mRNAs in radioresistant RS-FaDu cells derived from FaDu cells. Through validation experiments and subsequent preliminary investigation, TCONS_00018436 emerged as a promising candidate for studying the molecular mechanism underlying radioresistance of HSCC. Moreover, a large number of lncRNAs or mRNAs still awaits for being discovered through the bioinformatics analyses. In conclusion, our data laid the foundation for further investigating the roles of these lncRNAs and mRNAs in the occurrence and development of HSCC radioresistance. In addition, novel therapeutic targets and diagnostic biomarkers are likely to be identified in the future on the basis of our data.

## MATERIALS AND METHODS

### Establishment of a radioresistant cell line

The HSCC FaDu cell line was purchased from the Type Culture Collection of the Chinese Academy of Sciences (Beijing, China). The cells were cultured in MEM (Gibico, Grand Island, NY, USA) with 10% fetal bovine serum (FBS), 2 mM glutamine, 100 units/ml penicillin, and 100 μg/ml streptomycin and incubated at 37°C with humidified 5% CO_2_.

RS-FaDu cells were created by repeatedly exposing the parental FaDu cells to irradiation [15]. Briefly, FaDu cells were grown in 75-mm^2^ cell culture plates. After the cells reached 70–80% confluence, they were irradiated with X-rays at room temperature. The X-ray generator (MBR-1505R; Hitachi Medical Co., Tokyo, Japan) was operated at 210 kV and 10 mA, with 0.5 mm Al external filtration. The dose rate was 1.8 Gy/min. The cells were exposed to doses of 2, 4, 6, 8, and 10 Gy and were irradiated with each dose twice (total dose of 60 Gy). An interval of 2 to 4 weeks between each dose allowed the surviving cells to regenerate. The process of irradiation and culture lasted for about 10 months. The HSCC cell clones that recovered after exposure to ionizing radiation were collected for further experiments.

### Patient specimens

Primary and recurrent tumor samples were obtained from 13 patients who received radiotherapy followed by surgery in Qilu hospital from March 2013 to October 2015, and then salvage surgery due to local recurrence. After surgery, samples were cleaned with phosphate-buffered saline (PBS) and immediately put into liquid nitrogen at once. At least 24 h later, samples were transferred to −80°C for long-term storage. Characteristics of patients were summarized in Table 6. Prior to this study, written informed contents were signed, and this study was conducted under the approval of the institutional review board of the Ethics Boards of Qilu Hospital.

**Table 6:** Clinical characteristics of patients

Characteristics	No. Patients
Sex	
Female	0
Male	13
Age (years old)	(Median 57, Range 49–67)
Drinking	
Regularly	9
Occasionally	4
Seldom	0
Smoking	
Regularly	10
Occasionally	0
Seldom	3
*Histological Differentiation	
Well-Moderate	2
Poor	11
*Clinical Stage	
I + II	1
III + IV	12
*Treatment	
S + X	13

* at the first visit.

S: Surgery; X: radiation.

### Clonogenic assay

Both parental FaDu and radioresistant RS-FaDu cells were plated in six-well culture plates and irradiated with a single dose of 0, 2, 4, or 6 Gy, respectively. Plated cell numbers were as follows: 300 cells for 0 Gy, 600 cells for 2 Gy, 900 cells for 4 Gy, and 1200 cells for 6 Gy. Following irradiation, the cells were cultured in a 5% CO_2_ atmosphere at 37°C, and the medium was changed every 3 days. After 12 days, colonies were fixed with ethanol for 15 min and stained with 0.1% crystal violet for 15 min. Colonies with > 50 cells were scored with a ColCount colony counter (Oxford Optronix, Oxford, United Kingdom). All experiments were performed in triplicate. The survival fraction (SF) was estimated by the following formula: SF = [number of colonies formed/number of cells seeded × plating efficiency of the control group], where plating efficiency was calculated as the ratio between colonies observed and number of cells plated. Dose-response clonogenic survival curves were plotted on a log-linear scale using Graphpad Prism 5 software.

### Apoptosis assay

Apoptotic cells were identified by using the Annexin V-FITC and propidium iodide (PI) apoptosis detection kit (BestBio, Shanghai, China) according to the manufacturer's instructions. Briefly, RS-FaDu and FaDu cells were seeded at a density of 4 × 10^5^ cells in six-well plates and were incubated for 12 h before being treated with 4 Gy of radiation. The cells were then harvested at four different time points after X-ray exposure (0, 24, 48, and 72 h, respectively). After being washed twice with PBS, the cells were resuspended in 400 μL 1 × binding buffer and stained with 5 μL Annexin V-FITC for 15 min and 10 μL PI for 5 min at 4°C in the dark. Apoptosis was analyzed by a Gallios flow cytometer (Beckman Coulter, Brea, CA, USA). The percentage of total apoptosis was calculated as the sum of the early apoptosis (Annexin V+/PI−) and the late apoptosis (Annexin V+/PI+). The experiments were repeated three times and data were analyzed by Kaluza software (version 1.2; Beckman Coulter).

### RNA extraction and microarray analysis

RS-FaDu and FaDu cells were seeded at a density of 4 × 10^5^ cells in three six-well plates each. They were treated with 4 Gy of radiation and then cultured under the indicated experimental conditions. Cells were harvested at three different time points after X-ray exposure (0, 2, and 48 h, respectively). Total RNA from each sample was extracted using TRIzol reagent (Invitrogen, Carlsbad, CA, USA) following the manufacturer's instructions. RNA concentration was quantified by the NanoDrop ND-1000 (NanoDrop Technologies/Thermo Scientific, Wilmington, DE, USA), and RNA integrity was assessed by standard denaturing agarose gel electrophoresis. The sample preparation and microarray hybridization were performed based on the manufacturer's standard protocols with minor modifications. Briefly, total RNA was purified after removal of rRNA and tRNA (mRNA-ONLY^™^ Eukaryotic mRNA Isolation Kit, Epicentre, Madison, WI, USA). Then, each sample was amplified and transcribed into fluorescent cRNA, along the entire length of the transcript without 3’ bias, utilizing a random priming method. The labeled cRNAs were hybridized onto the Agilent Human LncRNA v4.0 (4 × 180 K, Arraystar; Agilent Technologies, Santa Clara, CA, USA). The slides were then washed, and the tiff-format original array images were acquired by the Agilent G2505C Scanner (Agilent Technologies).

### Microarray data analysis

The tiff-format original array images were pre-processed via Agilent Feature Extraction software (version 11.0.1.1) and then quantile normalization and differential expression analysis were conducted using the GeneSpring GX software package (version 11.5.1; Agilent Technologies). Cluster analysis and graphical illustration were performed using Cluster 3.0 software. Time-course differentially expressed lncRNAs and mRNAs with statistical significance between the two groups were identified through scatter plot filtering and volcano plot filtering. To filter out outlier samples, we performed hierarchical clustering to show any differences in expression intensity between the clustering group and true group results. The differentially expressed mRNAs were submitted to six pathway databases (KEGG PATHWAY, PID Curated, PID BioCarta, PID Reactome, BioCyc, Reactome, and Panther) for pathway enrichment analysis. After lncRNA and mRNA correlation, lncRNA target prediction included cis-prediction and trans-prediction. To determine cis-prediction, we searched for mRNAs that were in the region of 10 kb around the lncRNA. Trans-prediction was based on sequence alignment, which aligns lncRNA to the 3′UTR of mRNA. Then lncRNA-mRNA pairs that share similar sequences were identified from the trans-prediction results. In the lncRNA target prediction analysis, mRNA targets were predicted from cis-prediction and trans-prediction.

### Validation of lncRNA and mRNA expression by quantitative real-time polymerase chain reaction

Quantitative real-time polymerase chain reaction (qRT-PCR) was used to validate the microarray data. Briefly, total RNA was reverse-transcribed to cDNA using SuperScript^™^ III Reverse Transcriptase (Invitrogen) following the manufacturer's protocol. qRT-PCR was performed using the SYBR Green chemistry in the GeneAmp PCR System 9700 (ABI Applied Biosystems, Foster City, CA, USA). The forward and reverse primers for validation are listed in Table 7. PCR was performed in a 10-μL reaction volume and consisted of an initial denaturation step at 95°C for 10 min followed by amplification with 40 cycles at 95°C for 10 sec and 60°C for 60 sec. The threshold cycle (CT) was defined as the cycle number at which the fluorescence passed a predetermined threshold. Both target and reference (β-actin) genes were amplified in separate wells in triplicate. Gene expression was calculated using the comparative threshold cycle (2^−ΔCT^) method.

**Table 7:** Primers used for qRT-PCR

mRNAs/lncRNAs	Forward primers (5′–3′)	Reverse primers (5′–3′)	bp
ENST00000470135	TTGCCAGCAATTCATCAGAG	GGGATATGCCAACCTTGAGA	151
TCONS_00010875	TCGTTCACACACCCACTCAT	CGAGTGGGCAAGTTAGTGTG	153
TCONS_00018436	CCACCTCAGGATGGAAATGT	TCCCCAACCAAAGTCTTGTC	160
hox-HOXD10-35	GCTCCTTCACCACCAACATT	AAATATCCAGGGACGGGAAC	154
CKMT1A	ACCTGACCCCAGCAGTCTAT	AACACGTTCCACCTCTCGTC	374
GPNMB	AAGATTGCCACTTGATGCCG	TCCCTCATGTAAGCAGAAGGTC	75
FBLN5	CTCACTGTTACCATTCTGGCTC	GACTGGCGATCCAGGTCAAAG	89
GDA	GCTGGAAGTAGCATAGACCTGC	TCTTCTGCAAAGTCGATGTTCTG	95
ACTB	GTGGCCGAGGACTTTGATTG	CCTGTAACAACGCATCTCATATT	73

### Lentiviral transfection assay

Lentivirus containing short hairpin RNA (shRNA) of TCONS_00018436 or empty vectors used as control were purchased from GeneChem (Shanghai, China). And the lentiviral transfection assays were performed following manufacture's instructions. Stably transfected cells were screened by Puromycin (3 mg/mL) purchased from Sigma.

### Statistical analysis

Data were presented as mean ± standard deviation (SD) and statistical differences between two experimental groups were determined by using paired *t*-test or Student's *t*-test on SPSS 17.0 software (SPSS Inc., Chicago, IL, USA). Statistical differences in the microarray results were analyzed by fold change (FC) and *P*-value, and the FC and *P*-value were calculated based on normalized data. FC was calculated by computing the ratio of mean intensity of the case group to that of the control group, while *P*-value was calculated using Student's *t*-test. The thresholds for differentially expressed genes were set at FC ≥ 2.0 and *P*-value < 0.05. For the lncRNA and mRNA correlation analysis, the Pearson correlation coefficient was calculated to show the correlation between lncRNA and mRNA expression and *P*-value was calculated to show the significance of the Pearson correlation coefficient. Correlation > 0.99 or correlation < −0.99, and *P*-value < 0.05 were adopted to filter out random relationship. In all analyses, a two-sided *P*-value < 0.05 was considered statistically significant.

## SUPPLEMENTARY MATERIALS FIGURES AND TABLES













## Abbreviations

HSCC: hypopharyngeal squamous cell carcinoma
CRT: concomitant chemoradiotherapy
IMRT: intensity-modulated radiotherapy
IGRT: image-guided radiotherapy
TOMO: helical tomotherapy
lncRNA: long noncoding RNA
qRT-PCR: quantitative real-time polymerase chain reaction
GO: gene ontology
