# Supplementary material for: Time-course differential lncRNA and mRNA expressions in radioresistant hypopharyngeal cancer cells
Source: Oncotarget. 2017 Apr 21;8(25):40994–1010. doi: 10.18632/oncotarget.17343 (PMC5522212; doi:10.18632/oncotarget.17343)
Supplement: Supplementary file 1 [file oncotarget-08-40994-s001.pdf]

# Time-course differential lncRNA and mRNA expressions in radioresistant hypopharyngeal cancer cells

## Supplementary Materials

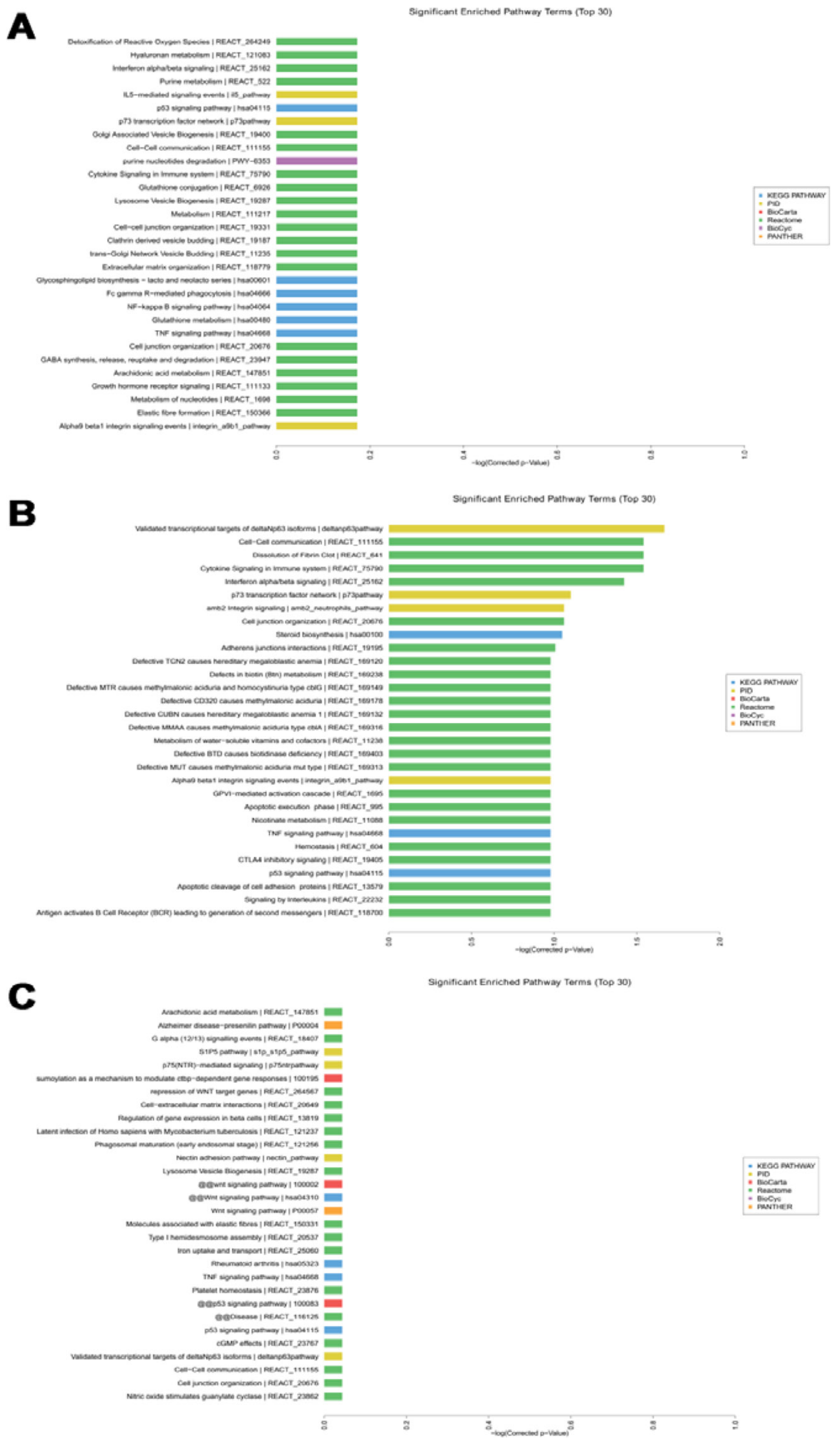


**Supplementary Figure 1: Top 30 significant enriched pathway terms.** In the figure, more significant term associates with larger –log(Corrected *p*-value) and different colors stand for different pathway databases. FaDu-RS vs. FaDu. (**A**): 0 h, (**B**): 2 h, (**C**): 48 h.

**Supplementary Table 1: Common differentially upregulated and downregulated lncRNAs in RS-FaDu cells at 0, 2, and 48 h after 4 Gy radiation, respectively.** See Supplementary_Table_1.

**Supplementary Table 2: Common differentially upregulated and downregulated mRNAs in RS-FaDu cells at 0, 2, and 48 h after 4 Gy radiation, respectively.** See Supplementary_Table_2.

**Supplementary Table 3: Significantly enriched pathway terms were presented after functionally annotating differentially expressed mRNAs in FaDu vs. RS-FaDu cells at 0 h after irradiation.** See Supplementary_Table_3.

**Supplementary Table 4: Significantly enriched pathway terms were presented after functionally annotating differentially expressed mRNAs in FaDu vs. RS-FaDu cells at 24 h after irradiation.** See Supplementary_Table_4.

**Supplementary Table 5: Significantly enriched pathway terms were presented after functionally annotating differentially expressed mRNAs in FaDu vs. RS-FaDu cells at 48 h after irradiation.** See Supplementary_Table_5.
